# Indigenous Adolescents’ Perception of an eMental Health Program (SPARX): Exploratory Qualitative Assessment

**DOI:** 10.2196/games.8752

**Published:** 2018-07-05

**Authors:** Matthew Shepherd, Sally Merry, Ian Lambie, Andrew Thompson

**Affiliations:** ^1^ School of Counselling, Human Services and Social Work Faculty of Education The University of Auckland Auckland New Zealand; ^2^ Ngāti Tama, Māori tribe Taranaki area New Zealand; ^3^ Department of Psychological Medicine School of Medicine The University of Auckland Auckland New Zealand; ^4^ School of Psychology Faculty of Science The University of Auckland Auckland New Zealand

**Keywords:** Māori youth, indigenous, depression, computerized CBT, consumer views, serious games, virtual worlds

## Abstract

**Background:**

Depression is a major health issue for indigenous adolescents, yet there is little research conducted about the efficacy and development of psychological interventions for these populations. In New Zealand there is little known about taitamariki (Māori adolescent) opinions regarding the development and effectiveness of psychological interventions, let alone computerized cognitive behavioral therapy. SPARX (Smart, Positive, Active, Realistic, X-factor thoughts) is a computerized intervention developed in New Zealand to treat mild-to-moderate depression in young people. Users are engaged in a virtual 3D environment where they must complete missions to progress to the next level. In each level there are challenges and puzzles to completeIt was designed to appeal to all young people in New Zealand and incorporates several images and concepts that are specifically Māori.

**Objective:**

The aim was to conduct an exploratory qualitative study of Māori adolescents’ opinions about the SPARX program. This is a follow-up to an earlier study where taitamariki opinions were gathered to inform the design of a computerized cognitive behavior therapy program.

**Methods:**

Taitamariki were interviewed using a semistructured interview once they had completed work with the SPARX resource. Six participants agreed to complete the interview; the interviews ranged from 10 to 30 minutes.

**Results:**

Taitamariki participating in the interviews found SPARX to be helpful. The Māori designs from the SPARX game were appropriate and useful, and the ability to customize the SPARX characters with Māori designs was beneficial and appeared to enhance cultural identity. These helped young people to feel engaged with SPARX which, in turn, assisted with the acquisition of relaxation and cognitive restructuring skills. Overall, using SPARX led to improved mood and increased levels of hope for the participants. In some instances, SPARX was used by wider whānau (Māori word for family) members with reported beneficial effect.

**Conclusions:**

Overall, this small group of Māori adolescents reported that cultural designs made it easier for them to engage with SPARX, which, in turn, led to an improvement in their mood and gave them hope. Further research is needed about how SPARX could be best used to support the families of these young people.

## Introduction

### Global Understanding of Depression

Depression is a major contributor to poor health [1] and is a global concern, with lifetime prevalence rates of depression ranging from 15% to as high as 25% [2]. Depression is common among children and adolescents, with community studies showing reported rates of 4-7.5% in international adolescent populations [3,4]. Depression in adolescence can persist into adulthood and has a variety of adverse psychiatric and psychosocial consequences (eg, poor academic performance, social dysfunction and substance abuse) [5-11].

### Māori Experience of Depression

In New Zealand (NZ) mental illness has long been identified as one of the most significant threats to the health status of all young people, with Māori an important indigenous group within the population [12,13]. A New Zealand–representative study showed that rates of depression for Māori high school students (at 13.9%) were comparable to young European New Zealanders (12.1%), with 18.3% of Māori girls and 8.7% of Māori boys reporting depressive symptoms in the clinical range on the Reynolds Adolescent Depression Scale-Short Form (RADS-SF) [14]. With low rates of access to mental health services reported for all groups (particularly for taitamariki) and with the rising numbers of young Māori in New Zealand [14], it is important that mental health resources are available to them.

### Cognitive Behavioral Therapy

The National Institute for Clinical Excellence recommends cognitive behavioral therapy (CBT) as the preferred psychological therapy for treating mild to moderate depression in children and adolescents [15] with emerging evidence that computerized CBT (cCBT) can be as effective as treatment as usual in the reduction of depressive symptoms in adolescents [16,17,18,19] and superior to waitlist control [20].

### Cognitive Behavioral Therapy and Māori

CBT in relation to ethnic minority groups has been criticized because of its perceived lack of relevance to some of these groups [21]. CBT foundations are firmly grounded in a scientific view of the world (the importance of rational thinking and seeking objective evidence), which has led to questions about the efficacy of CBT for clients with more spiritually based beliefs [22,23].

There is little evidence to guide practice as ethnic minority groups are largely missing from efficacy studies that make up the evidence base for psychological treatments [24-26], particularly studies that focus on adolescents and children. Horrell examined the effectiveness of CBT with adult ethnic minority clients and demonstrated that, based on 12 studies, CBT appears to be an effective treatment for use with clients from ethnic minority backgrounds [27]. Seven of the 12 studies demonstrated significant treatment gains with CBT compared with a placebo or wait-list control. CBT was effective in reducing a range of symptoms such as Depression, Posttraumatic Stress Disorder, Generalized Anxiety Disorder and Panic Disorder. The authors recommend that further research needs to be conducted to determine whether CBT is a consistently effective intervention for ethnic minority groups [27].

Similarly, there is little published research regarding psychological interventions for Māori [28-30] and the attitudes and opinions of tangata whenua (indigenous people of New Zealand) towards the development of a CBT program [28]. Ideally, clinicians should provide evidence-based care to ethnic minority populations that has been tailored to make it sensitive and more acceptable to the culture of the individual receiving treatment [25].

### Access to e-Therapies for Indigenous Young People

Adolescent ethnic minority groups have lower access rates when seeking professional treatment for depression [31-33]. Therefore, more needs to be done to facilitate easier access to treatments. With the proliferation in the use of technology there has been increased interest in the potential for cCBT to be used as a low-cost, easily accessible option for those in need of treatment [18]. It is thought cCBT has the potential to increase access to therapy for these indigenous youths if it can be delivered in a way that is acceptable and appealing to these young people. For example, cCBT can be customized to the end-user, the client’s data can be easily accessed (with permission) and access to computers can reduce the cost of therapy [17]. In New Zealand, there are low access rates to mental health services by young people [34], cCBT has the potential to provide access to a therapeutic intervention, particularly for those people who are not accessing current services. The opinions and the attitudes of ethnic minority tangata whenua in relation to the development of a cCBT resource have only begun to be captured [35]. This study highlights the results of the user feedback from taitamariki in New Zealand who completed SPARX. SPARX is a cCBT program that teaches young people (12-19 years old) CBT skills to better manage their mild to moderate depressive symptoms. There are seven modules contained within the SPARX program and each module takes 20-30 minutes to complete. Users are engaged in a 3D environment where they must complete missions to progress to the next level. In each level there are challenges and puzzles to complete, one example is that the user must shoot down the Gloomy Negative Automatic thoughts. SPARX includes features that assist with the engagement of taitamariki. For example, SPARX contains an in-game environment that is particularly unique to New Zealand, there are Māori words used in SPARX, and there are Māori objects placed throughout SPARX, such as waka (canoe). There is also the ability to customize one’s avatar to represent what a Māori person may look like to the participant. All these factors aim to increase the applicability of SPARX for Māori. For further information about SPARX please refer to the study by Merry et al [19].

### Aims

The aim of this study was to follow up on a previous study that investigated taitamariki opinions in the process of codesigning the development of a beta version of SPARX [35]. This current study summarizes taitamariki experiences of completing the developed SPARX program.

## Methods

Ethics approval was granted by the Northern Regional Y committee (NTY 2009/01/03) of the New Zealand Ministry of Health. Consent was obtained from all participants and parental consent was sought for those under the age of 16 years. No inducements were offered.

### Epistemological and Methodological Considerations

The first author (MS) conducted all the interviews and took a lead in analyzing this data. MS holds a critical realist position, having Māori whakapapa (genealogy) and was aware of following an indigenous process when collecting and interpreting the data [36].

### Kaupapa Māori Methodology

Over the past three decades there has been an increasing awareness about the need to acknowledge Māori epistemology coupled with Māori ways of conducting research and, hence, the term “Kaupapa Māori research” was developed [37]. Western research traditionally holds an individualistic approach to epistemology. Māori healing practices differ to western approaches in that Māori will use karakia (prayer) as a key component to healing [38]. This highlights the reliance and incorporation of a God (Atua) or many deities. Accessing the spiritual dimension (wairua) for general wellbeing is an important dynamic in Māori life. Western psychological models such as CBT have tended to focus on an individual’s internal psychological state, for example, a change in one’s thoughts and feelings, which leads to improved mental health. However, the traditional Māori perspective has been to view the world in a collectivist way. Māori culture places an emphasis on the individual acting in a way that would seek to put whānau (extended family), and iwi (tribe) needs before individual needs. Therefore, western therapeutic approaches might have limited appeal or limited therapeutic power with Māori young people. With its analytic focus on individual thoughts, behaviors, and feelings, cCBT without processes of cultural connection might be considered antithetical to Māori worldviews. Alternatively, the use of Māori images, the use of story-telling, and opportunities for holistic learning processes embedded in an intervention may have some appeal. This has implications for research from a Māori worldview and this way of carrying out studies has come to be known as Kaupapa Māori research [37,38,39,40]. Since Kaupapa Māori research includes methodology, epistemology, theory, Māori tikanga (customs and protocols), MS was mindful about incorporating these aspects into this study. The observation of Māori protocol was important when meeting with the participants and is outlined in more detail below.

#### Recruitment of Participants

Two schools within the wider Auckland, New Zealand, area were asked if some of their students could be approached to participate in the study. These schools were chosen because they expressed interest in the study, had the necessary infrastructure, and had relatively large Māori populations. Participants for this study had accepted an invitation by school guidance counsellors to complete an open trial of SPARX and seven were screened for depression using the patient health questionnaire. Those in the mild to moderate range with scores of 10 to 19 inclusive and at low risk of self-harm were invited to complete baseline assessments, which included the Child Depression Rating Scale-Revised version (CDRS-R).

### Data Collection

Once the participants had completed SPARX, all seven were asked to complete an interview which utilized a semistructured interview schedule (Multimedia Appendix 1). One participant declined the interview. Mihi whakatau (culturally formal method of beginning a meeting), karakia (prayers), and kai (food) were included in the interview process to incorporate a Kaupapa Māori methodology. All participants had completed all seven levels of the SPARX program. The topics of the questions included whether SPARX was helpful, whether they thought their mood had improved, what taitamariki thought about the content of the cCBT program, and which skills they found helpful. Opinions were sought regarding the main characters and the Māori designs in the SPARX program and the computer control mechanism used to move the main character. MS also explored whether whānau had supported taitamariki in their use of the SPARX resource. MS conducted all the interviews, which lasted approximately 30 minutes.

### Qualitative Data Analysis

An inductive approach was incorporated while following Braun and Clarke’s [41] six-step process of thematic analysis, which was used to identify, analyze, and present the main themes from the data. The participant interviews were transcribed and read through by MS to gain an initial understanding of the themes emerging from the data. The transcripts were then loaded into a computer software program (NVivo8).

Initial codes were generated during the first reading of the data and codes that were similar but distinct were kept separate. A second reading of the data confirmed the coding of the themes. To ensure reliability, another researcher read one-third of the transcripts and their themes were compared to the themes found by MS. Differing opinions about themes were discussed and an agreement was reached about which themes to include.

## Results

### Overview

The mean age of the participants was 14.67 years. Five of the participants were female (aged 14-16) and one participant was male (age 14). All the participants self-identified as being Māori. All of the participants in this study were part of a previous study [42], in which their depression symptoms were measured and monitored using the CDRS-R. A summary of scores at the three time points showed a reduction in depressive symptoms after completion of SPARX. The baseline mean score on the CDRS-R was 49.43 (SD 9.86), posttreatment 2-month mean score was 26.86 (SD 10.56), and the follow up 5-month mean score was 31.71 (SD 20). This was significant *t* (7)=3.930, *P=*.008 and effect size=1.49. The improvement was maintained at follow-up although the change from baseline was not as large as the change from post treatment *t* (7)=2.56, *P=*.043 and effect size=0.97. The score however dropped to a mean that was within the normal range. Six young people were interviewed via a semistructured interview process. A number of themes emerged from the data and were organized into five categories as shown in Table 1.

### SPARX is Helpful as it Taught CBT Skills

All of the participants (N=6) who were interviewed described SPARX as being helpful. They stated that SPARX gave good advice and that it teaches real-life techniques and skills. The participants described skills such as relaxation and cognitive restructuring as being easy to use in everyday life. Taitamariki appeared surprised that the breathing techniques were especially beneficial for them.

Well, I have been feeling down sometimes and I have used the breathing techniques and all that type of stuff and it was actually really helpful.

Female participant (P)1

It was really good and helpful. Like when I needed to calm down using the technique, it actually helped a lot. Yes, so overall it was pretty good, the breathing in out one. At first, I thought it was a bit weird and I was like ‘oh, my god, this is not going to work’ but by just taking my time and working through it, it actually helped me.

Female participant P2

Three of the participants were able to give examples of how cognitive restructuring helped them, while also reporting that this was one of the more difficult concepts to understand.

Like when my Mum and Dad split up I thought it was my fault and I could have done something. And then after I did SPARX I did a lot of thinking and I couldn’t have done anything because they weren’t happy, and it was out of my control. So it helps me understand.

Female P1

Well, if I was thinking something negative, yes, I would go out for a walk and I would think about it until it became positive.

Female P2

It was like fun because of the game and it helped me with problems and solving. It was very good, it taught me not to think violent, just talk. This girl one of my cousin’s friends, went to my house with my cousin and they were rude to my Mum. My Mum said, “tell her to come and say sorry.” And I scooped her up and told her to say sorry. Oh no, I snapped down and told her I was going to smash her and then I thought about it and I said “Naw, that no good.” Just go and say sorry to my Mum and it will be all good.

Female P3

Taitamariki were able to articulate how SPARX had helped to improve not just their mood but also their life and significant relationships:

It was actually really helpful. I had a lot of problems and it did help me a lot. I felt much more better and it taught me a lot of lessons. I am actually enjoying life now and I am still going through my up and downs, but it is a bit better. I am more happier and I don’t feel down as much as I used to.

Female P1

But then I actually thought about it and I have actually come a long way from where I was. And SPARX had a big part of that, so that helped. When I first started it I was always sad, like practically every day, but now I am like happy and hang out with my friends and enjoy myself. Most days I am happy.

Female P2

The taitamariki were able to reflect on how it had helped their whānau. Some spoke about how they would talk to a sibling or a parental figure and suggest to them that they should play SPARX. Participants reported that little persuasion was needed for other whānau members to use SPARX.

**Table 1:** Categories and themes from analysis (N=6).

Categories	Themes
SPARX is helpful as it taught Cognitive Behavioural Therapy skills (N=6)	SPARX was able to teach relaxation skills (N=4) SPARX was able to teach cognitive restructuring skills (N=3) SPARX helped improve mood and quality of life (N=3) SPARX was able to help whānau members (N=3) SPARX was able to increase participants’ level of hope (N=2)
Māori designs assisted with the engagement of Māori adolescents (N=5)	Māori designs were appropriate and appreciated (N=5) The ability to customize the characters with Māori designs was beneficial (N=2) Having Māori designs increased cultural identity (N=2)
Characters in SPARX provided hope and helpful advice (N=4)	Characters were helpful (N=3) The Guide was easy to understand (N=4) The Bird of Hope character was helpful (N=2)
SPARX Game Design was enjoyable and provided challenging factors (N=3)	Character control mechanism worked well (N=3) Participants found the puzzles to be difficult (N=3)
SPARX Booklet was useful to record participants’ thoughts and feelings (N=2)	The booklet is a useful resource (N=2)

Well, like during the holidays me and my Mum are going to do it together because she is going through a lot of stress as well. We would both sit down and do it and sort it out. I told Mum about the thing [SPARX] and she said that she would like to try it. I am hoping it will actually help her out and take less off her shoulders.

Female P1

I think my Mum. She has noticed the change as well and we have actually both put in the effort and things have been improving a lot more. We always used to fight and it used to be like if you are not going to change, why should I bother. So I just decided to make a change and ever since then my Mum has noticed and she has been nicer and she is actually putting in a good effort. I think SPARX has helped a lot in a way and really it was just up to me and my Mum but applying those skills actually helped. So that is a big part.

Female P2

Some taitamariki responded well to the psycho-education component in SPARX about having “hope.” They reported that their mood had improved by being able to receive encouragement and adopt the new idea that life can be better if you have hope.

Like how they [characters in SPARX] always give you advice. Like for the hope one, that was really good. Like there is always hope and stuff.

Female P1

When I first started SPARX I was still feeling low and that but now that I have finished SPARX it has made me a bit happier and feeling all right. That my future is not going to be a negative future. It is going to be a positive future.

Female P2

### Māori Designs Assisted With the Engagement of Māori Taitamariki

The majority of taitamariki (N=5) found the designs to be appropriate and felt the designs assisted them to engage with SPARX. The ability to customize their character helped to ensure this. Some taitamariki stated openly that the Māori designs helped to increase their Māori identity.

That is cool. It is different from other games, it can be fun, but it also has stuff from your culture and things you can relate to. So that was better.

Female P1

Yes, I think it does help because it is different from other games, they don’t have anything like Māori culture. It is like unusual to see that kind of stuff in games. But having that makes it easier to relate to.

Female P2

Some taitamariki appreciated the ability to personalize their SPARX character with Māori designs on the clothing.

That was pretty cool [the customization process], but I think there should be more stuff because it is only like a couple of haircuts and skin tones and all that type of stuff.

Female P1

I got to make her look however I wanted, and I was able to choose the clothing and the hair style and that is really fun.

Female P2

Some taitamariki clearly had their identity affirmed by the inclusion of Māori designs within SPARX.

I liked them. They made me feel like I was neat. It opened my Māori in me. It makes me feel like I’ve got Māori in me and that everyone knows who I am.

Female P1

It is really cool. It is good to do something that has part of your heritage in it.

Female P2

### Characters in SPARX Provided Hope and Helpful Advice

Taitamariki were able to describe how characters like the Guide, Mentor and Bird of Hope were all able to provide support and psychological education that was both helpful and hopeful.

Some taitamariki identified that it was beneficial to have nonplaying characters in SPARX. This provided an opportunity for taitamariki to practice the skills that they had been learning within SPARX.

It was pretty cool that you got to practice skills on them [nonplaying characters]. Like how do you make a win-win conversation and stuff, so that was pretty good.

Female P1

The lady [Mentor], those people that helped me out when I got stuck.

Female P2

Taitamariki identified strongly with the Guide, particularly because of the way he looked and sounded. This was clearly important for the Māori participants and enhanced their ability to relate to the Guide. Taitamariki reported that they felt the Guide was communicating personally with them. The Guide was also able to reinforce the learning that took place within SPARX.

He [Guide] actually looked like a Māori and it was easier for me to relate to because he had the New Zealand accent and the Māori accent and stuff. Sometimes he would say a few things in Māori so that was really good. He would explain it and then afterwards he would break it down and make it easier for me to understand. That helped a lot.

Female P1

That was good. It helped me out because if I didn’t get what I was doing while I was actually doing it, it [the Guide] told me the proper meaning at the end.

Female P2

Some taitamariki benefited from having a character (the Bird of Hope) that embodied the concept of hope. Some taitamariki were encouraged by the function that the Bird of Hope provided.

It gave you information about what you are actually meant to do and how to handle these types of things, like thoughts.

Female P1

Oh, the bird is cute, hope. Yes, that was my little favorite thing on SPARKS is hope. Because she said, “I am always here to help you.” It was so cute and just the color of her. She looks so beautiful. I wish she was my pet.

Female P2

### SPARX Game Design was Enjoyable and Provided Challenging Factors

Taitamariki reported that, while SPARX seemed like a game that was fun, it was able to be helpful by giving advice as well. Taitamariki thought that SPARX could have been longer and one male participant thought that the puzzles could have been a lot harder, in contrast to the views of most female participants. There was some mixed opinion about whether the character control mechanism in SPARX was easy to use. Some taitamariki felt that the current way to control the character could have been improved by using the keyboard arrows (instead of the mouse) to move the character.

It was actually really good, yes. It was really easy, you just had to click, and it would move to where you clicked. That was pretty good.

Female P1

I was thinking instead of using the mouse you could use the keyboard thing. Yes, keyboard arrows, yes. The general control, it was just when I had to make her run I had to double click it and then she would run then she would stop. Yes. It was medium.

Female P2

Some taitamariki found the puzzles difficult, though no one stated that it put them off completing SPARX as only one taitamariki completed fewer than all seven levels. Taitamariki were also provided with the answers to the puzzles (in the booklet that accompanied SPARX) so that they could continue with SPARX if they got stuck.

The puzzles were kind of hard. The challenges were good, yes. When the pieces got burnt and I had to sort them out, I found that hard.

Female P1

SPARX could have been longer and harder, as I only found one of the challenges to be difficult.

Male P1

### SPARX Booklet was Useful to Record Participant’s Thoughts and Feelings.

The SPARX booklet was designed as a learning aid so taitamariki could record their thoughts and feelings while they were progressing through the seven levels of SPARX.

It was easier to write it down than to keep it inside. I would probably write more about my feelings down on a piece of paper than keep it inside me.

Female P1

## Discussion

### Principal Findings

In this small study we have identified the potential importance of cultural components of eMental health interventions and have sought to incorporate indigenous youths' opinions and thoughts about the beta version of SPARX. The taitamariki interviewed here provided feedback on the applicability of SPARX for taitamariki experiencing mild to moderate depression. The Māori designs were found to be appropriate and useful and the ability to customize the characters with Māori designs appeared beneficial, as this seemed to enhance cultural identity. Taitamariki found SPARX to be helpful because it was able to teach relaxation and cognitive restructuring skills. It helped improve participants’ mood and increased their levels of hope and, in some instances, was used by whānau members to good effect. The Guide was identified as Māori, and this appeared to increase engagement with the resource. In terms of the game design, the character control mechanism worked well. In general, participants found the puzzles to be difficult, while the booklet that accompanied the resource was found to be useful by these young people.

Many themes that emerged from the present study have reinforced the themes from an earlier research study that investigated taitamariki opinions about the initial design of SPARX [35]. This includes feedback that SPARX could teach CBT skills and that SPARX was like a computer game that could help with depression. A dominant skill that was highlighted again was the breathing relaxation exercise. Once taitamariki had the chance to complete all seven levels, the other CBT skill they described most frequently was cognitive restructuring. These findings suggest that participants were able to learn CBT skills without the aid of a therapist which is in line with other findings [43]. It is interesting that these taitamariki were very similar to the held mainstream views of cCBT [18]. These results suggest that SPARX is acceptable to the indigenous people of New Zealand and has the potential to address some of the unmet mental health needs of taitamariki [14,44]. Its availability as a public health resource could assist with the issue of access rates for services to indigenous youth, as adolescents rarely seek treatment for depression [31,32,33].

The findings from an initial study [35] in relation to the Māori designs enhancing the engagement and gaming experience for taitamariki were confirmed with this study. This was important because culturally adapted mental health interventions targeted to a specific cultural group can be much more effective than interventions provided to groups from a variety of cultural backgrounds [45]. These findings suggest that, for Māori females (as most of the participants were female), SPARX did meet the aim of being culturally applicable. The finding from a previous study that some taitamariki participants appreciated the ability to customize their own playing character within SPARX was confirmed. Some taitamariki reported that Māori designs affirmed their Māori identity, and this appeared to enhance the well-being of taitamariki.

Shepherd et al [35] suggested that a whānau perspective could be incorporated into SPARX. Although we were unable to do this because of funding and time constraints, it is apparent from this study that taitamariki included some whānau members who were interested in completing SPARX. Taitamariki reported that, once whānau members became engaged in SPARX, this was helpful with one person even suggesting that it led to an increase in the quality of whānau relationships. Edwards and McCreanor [46] have highlighted the importance of whānau for Māori young people and the need for intervention programs to incorporate whānau.

### Strengths of this Study

This study conducted interviews with taitamariki who provided important information about their experiences in completing SPARX. Very few studies have collected information about indigenous youth user feedback of a cCBT intervention. The findings from this group were similar to themes identified by the 19 taitamariki who participated in an earlier study concerning SPARX and taitamariki [35]. This is effectively a co-design process with the incorporation of young people’s feedback on the beta version into subsequent iterations of SPARX.

### Limitations of the Study

This is a small study and is exploratory in nature, limiting generalizability. A convenience sample was used, and the number of participants was not determined by data saturation. There is potential that these findings did not uncover the full range of experiences with SPARX. Participants were mostly female; we were not able to explore whether we had responded adequately to comments made by male participants in the first study who wanted more physical activities, such as fighting; shooting creatures or other characters; or activities such as fishing and skateboarding. Some of these were incorporated but feedback on this was limited. The one male participant who did complete the follow-up interview reported that the puzzles and challenges within SPARX were too easy.

### Further Research

How SPARX could be used for whānau is an important area that warrants further research. The findings from this study suggest that some whānau may have gained psychological support serendipitously from the resource and that SPARX could play a role in strengthening whānau ora (family health). The concept of whānau ora is a New Zealand government strategy that is a part of *He Korowai Oranga: The Māori Health Strategy* [47]. This strategy highlights the importance of mental health and well-being for the overall health of Māori families.

### Practice Implications

Taitamariki reported that they were able to learn CBT skills from SPARX and to improve their mental health through this form of pedagogy. The Māori designs were appropriate and useful, and the ability to customize the characters with Māori designs was beneficial as this enhanced cultural identity. These opinions were expressed within the earlier study as well and point to the need for therapeutic interventions directed towards taitamariki to be culturally appropriate and relevant to them. It is likely that this holds true for other indigenous groups. It is important that a much larger study be conducted to explore the efficacy of SPARX on this cohort.

### Summary

The follow-up interviews from this study were important because they showed that the changes made resulting from feedback received from previous focus groups were mostly successful. Taitamariki found that SPARX helped improve mood and increased their levels of hope. Relaxation techniques were particularly helpful. SPARX was also able to help other whānau members. The Māori designs were appropriate and useful and the ability to customize the characters with Māori designs was beneficial as having Māori designs increased cultural identity connections. The characters in SPARX were helpful, for example, the Guide, which provided support and information that was easy to understand, and the Bird of Hope encouraged taitamariki to be hopeful.

## Appendix

Multimedia Appendix 1 Youth E-Therapy Follow Up Interviews – Interview Guidelines for Taitamariki.

## Abbreviations

cCBT: computerized cognitive behavioral therapy
CBT: cognitive behavioral therapy
NZ: New Zealand
SPARX: Smart, Positive, Active, Realistic, X-factor thoughts
