# Supplementary figures and images for: Indigenous Adolescents’ Perception of an eMental Health Program (SPARX): Exploratory Qualitative Assessment
Source: JMIR Serious Games. 2018 Jul 5;6(3):e13. doi: 10.2196/games.8752 (PMC6053605; doi:10.2196/games.8752)

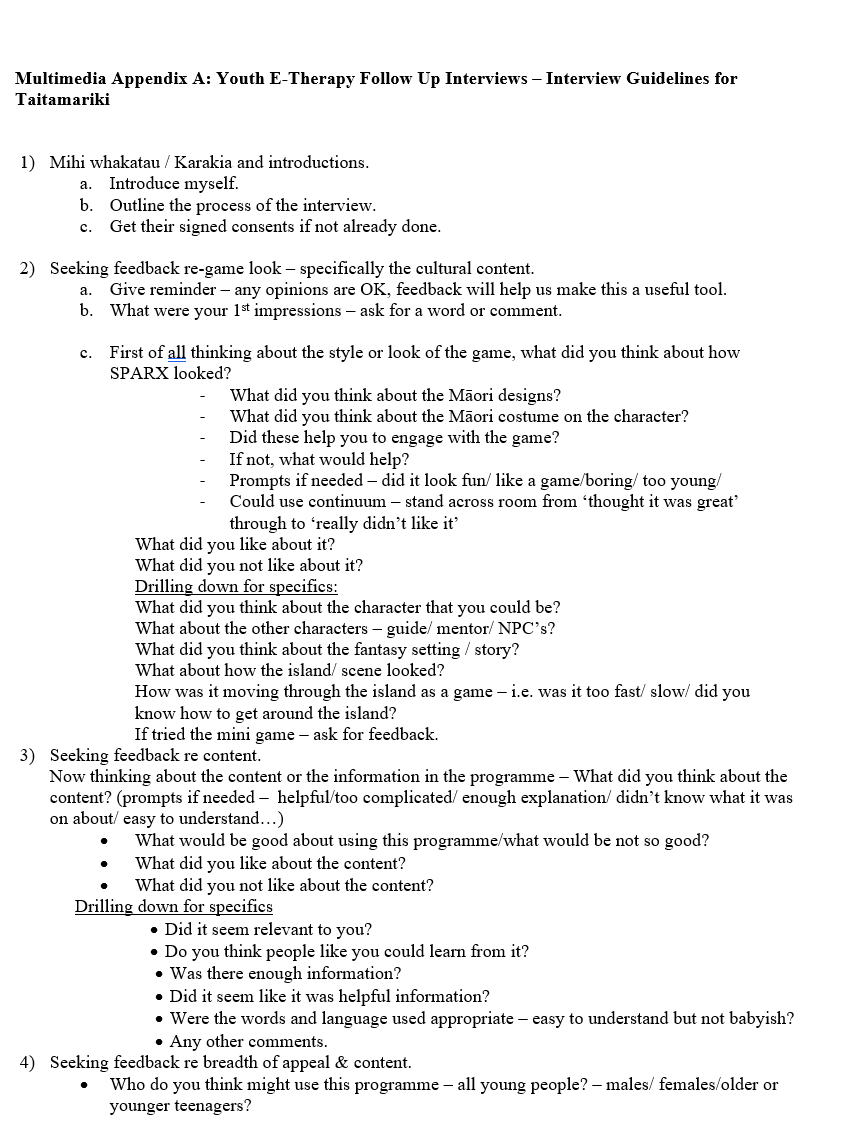

Supplement: Multimedia Appendix 1 [file games_v6i3e13_app1.png]
